# High expression of SULF1 is associated with adverse prognosis in breast cancer brain metastasis

**DOI:** 10.1002/ame2.12406

**Published:** 2024-04-08

**Authors:** Yitong Li, Tingting Feng, Qinghong Wang, Yue Wu, Jue Wang, Wenlong Zhang, Qi Kong

**Affiliations:** ^1^ NHC Key Laboratory of Human Disease Comparative Medicine, Beijing Engineering Research Center for Experimental Animal Models of Human Critical Diseases, Institute of Laboratory Animal Sciences Chinese Academy of Medical Sciences (CAMS) and Comparative Medicine Center, Peking Union Medical College (PUMC) Beijing China

**Keywords:** brain metastasis, breast cancer, potential therapeutic target, SULF1

## Abstract

**Background:**

Breast cancer is the most common cancer in women, and in advanced stages, it often metastasizes to the brain. However, research on the biological mechanisms of breast cancer brain metastasis and potential therapeutic targets are limited.

**Methods:**

Differential gene expression analysis (DEGs) for the datasets GSE43837 and GSE125989 from the GEO database was performed using online analysis tools such as GEO2R and Sangerbox. Further investigation related to SULF1 was conducted using online databases such as Kaplan–Meier Plotter and cBioPortal. Thus, expression levels, variations, associations with HER2, biological processes, and pathways involving SULF1 could be analyzed using UALCAN, cBioPortal, GEPIA2, and LinkedOmics databases. Moreover, the sensitivity of SULF1 to existing drugs was explored using drug databases such as RNAactDrug and CADSP.

**Results:**

High expression of SULF1 was associated with poor prognosis in advanced breast cancer brain metastasis and was positively correlated with the expression of HER2. In the metastatic breast cancer population, SULF1 ranked top among the 16 DEGs with the highest mutation rate, reaching 11%, primarily due to amplification. KEGG and GSEA analyses revealed that the genes co‐expressed with SULF1 were positively enriched in the ‘ECM‐receptor interaction’ gene set and negatively enriched in the ‘Ribosome’ gene set. Currently, docetaxel and vinorelbine can act as treatment options if the expression of SULF1 is high.

**Conclusions:**

This study, through bioinformatics analysis, unveiled SULF1 as a potential target for treating breast cancer brain metastasis (BM).

## Graphical abstract

Through mining public databases, up‐regulated and down‐regulated differential gene expression analysis on 70 patient samples was conducted. Through prognosis survival analysis and genetic variation analysis, SULF1 was identified as a core gene. Then, related biological mechanisms were explored by mining co‐expressed genes. Finally, combined clinical diagnosis and treatment potential drugs were explored.

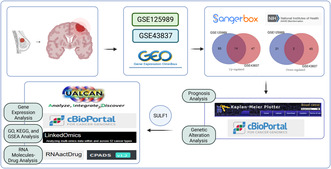

## 1 INTRODUCTION

Breast cancer (BRCA) is the commonest female malignant tumor worldwide.^1^ Typically it originates in the epithelium of the breast ducts.^2^ Based on histological and molecular evidence, BRCA can be clinically categorized into three subtypes: hormone receptor‐positive breast cancer (ER or PR positive), HER2 (ERBB2 positive) breast cancer, and triple‐negative breast cancer (TNBC) (ER‐/PR‐/HER2‐).^3^

Advanced‐stage BRCA exhibits extensive metastasis and is the second most common entity of metastases to the central nervous system.^4^ Studies indicate that 3%–10% of newly diagnosed breast cancers have metastasized by the time they are diagnosed.^5^ When tumors metastasize to the brain, they cause a range of corresponding symptoms, such as motor dysfunction, headaches, and seizures.^6^ It has been proven that the activity of late‐stage breast cancer treatment drugs like Trazumab, temozolomide, cis‐platinum, capecitabine, and paclitaxel is limited in the central nervous system due to the blood–brain barrier.^7^ Therefore, exploring the mechanisms and molecular complexity of BRCA with brain metastasis (BM) is crucial for better treatment, prognosis, and improved survival rates.

Previous studies have shown that breast cancer brain metastasis involves multiple steps, which include angiogenesis, invasion, intravasation, extravasation, and brain colonization.^8^ Multiple genes are involved and play essential roles in different steps of breast cancer brain metastasis. Among them, over‐expression of HER2 is an apparent risk factor for central nervous system metastasis.^9^ Additionally, blood–brain barrier disruption is a key factor in facilitating brain metastasis.^8^ However, the available findings have not yet demonstrated fully the molecular mechanisms of BM in BRCA.

SULF1 is a full‐length cDNA of a human protein with sulfatase activity and unique structural features. It plays a significant role in tumor proliferation, migration, invasion, and angiogenesis.^10^ Several studies suggest that SULF1 is associated with the proliferation and migration of cancer cells, which lead to poor prognosis in cancers such as ovarian cancer, chondrosarcoma, and colorectal cancer.^11^, ^12^, ^13^

Combined with transcriptome data, bioinformatic analysis has emerged in recent years as a fast and efficient way to identify dysregulated genes significantly associated with carcinogenesis, and may provide insights into potential therapeutic targets.^14^ Recent studies have explored predictive markers related to breast cancer prognosis by integrating transcriptome data, but there are few studies on the molecular mechanisms related to BM. This study collected data from the Gene Expression Omnibus (GEO) database, a comprehensive record of gene expression in the National Center for Biotechnology Information (NCBI) containing microarrays, next‐generation sequencing, and other high‐throughput functional genomics data forms, which are incorporated into existing studies.^15^ Based on GSE43837 and GSE125989 datasets, DEGs between primary breast cancer and brain metastatic breast cancer tissues were identified with the online software GEO2R. Thus, the intersection of up‐regulated and down‐regulated genes could be identified.

Further, overall survival (OS) and recurrence‐free survival (RFS) analyses were performed using KM‐Plotter for 16 common DEGs, and variations in these 16 genes were analyzed using the cBioPortal web tool. Genetic correlates of SULF1 were then explored through Gene Ontology (GO), the Kyoto Encyclopedia of Genes and Genomes (KEGG), and Gene Set Enrichment Analysis (GSEA). Finally, possible therapeutic drugs targeting SULF1 were investigated using the RNAactDrug and CADSP databases. Above all, SULF1 was identified as a gene up‐regulated explicitly in brain metastasis and a potential target for drug therapy with docetaxel and vinorelbine.

## 2 METHODS

### 2.1 Public database analysis

**FIGURE 1:**
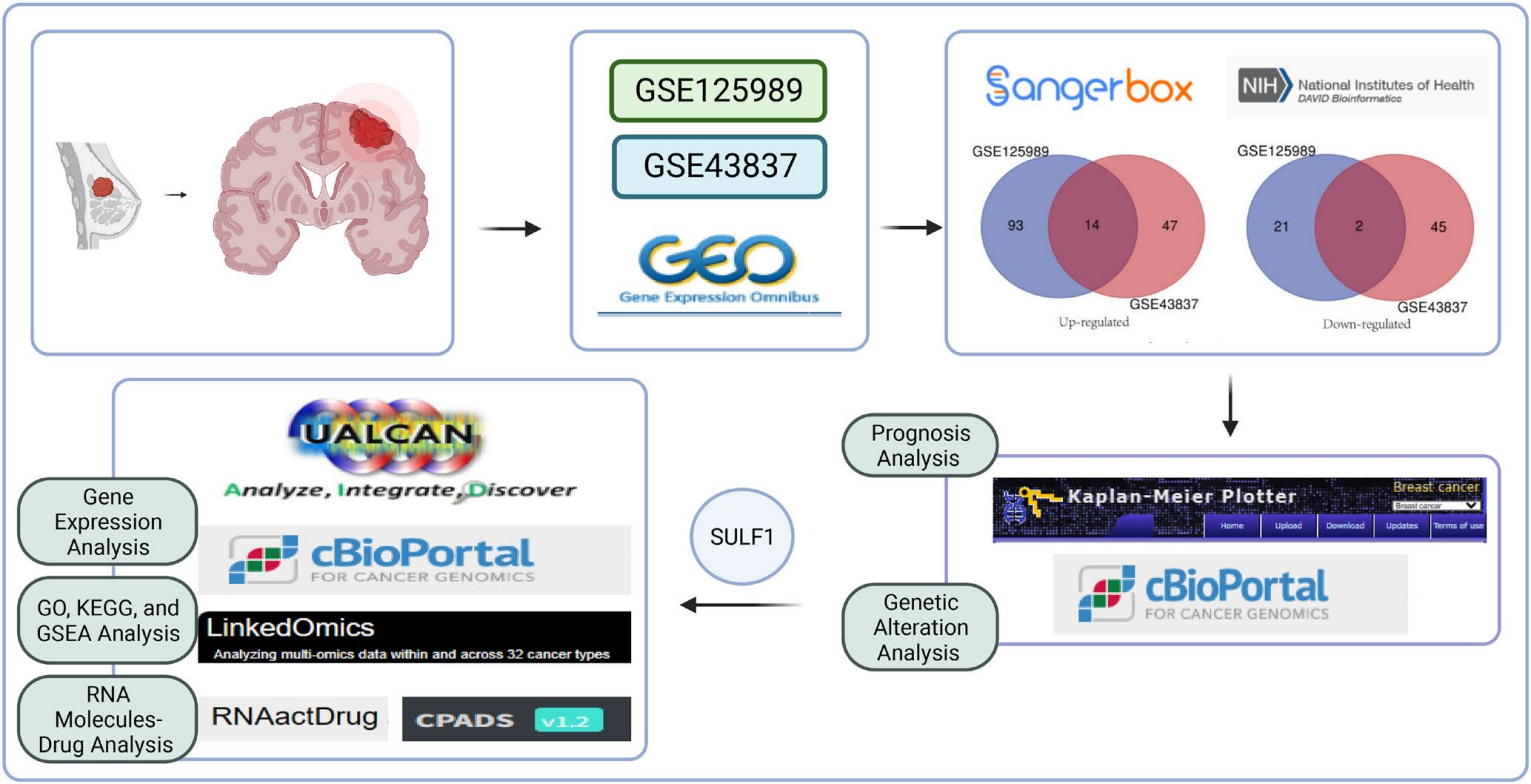
The methodological flowchart of this study. (Created with BioRender.com).

The gene expression datasets GSE43837 and GSE125989 were obtained from the GEO database (https://www.ncbi.nlm.nih.gov/geo/). The GSE43837 dataset consists of 38 clinical BRCA samples, containing 19 primary BRCA samples and 19 BM samples. The GSE125989 dataset includes 32 clinical BRCA samples, containing 16 primary BRCA samples and 16 BM samples. All the data were retrieved and available online, and this research did not involve any experiments on humans or animals. Furthermore, all informed consents had been obtained (Figure 1).

### 2.2 Data processing of DEGs

The GEO2R online analysis tool (https://www.ncbi.nlm.nih.gov/geo/geo2r/) detected the DEGs that were common to both primary BRCA and BM samples. The *p* value and |log_2_(fold change)| (|log_2_FC|) were calculated. Genes that meet the selection criteria, namely, a *p* value < 0.05 and |log_2_FC| ≥ 2, were considered DEGs. Statistical gene expression analyses were presented using a volcano plot established with the Sangerbox platform web tool (http://sangerbox.com/home.html). A Venn diagram presented the intersecting DEGs of 2 datasets analyzed with a web tool (https://bioinformatics.psb.ugent.be/webtools/Venn/).

### 2.3 Prognosis analysis

Kaplan–Meier survival analyses for overall survival (OS) and recurrence‐free survival (RFS) in BRCA patients were performed at the best cut‐off with the Kaplan–Meier Plotter web tool (http://www.kmplot.com).

### 2.4 Genetic alteration and expression analysis

The cBioPortal (https://www.cbioportal.org), an interactive online tool, was used to analyze the genetic alteration of 16 metastatic breast cancer DEGs of (INSERM, PLoS Med 2016). The ‘Cancer Types Summary’ module consisted of ‘alteration frequency,’ ‘mutation data,’ and ‘copy number alteration (CNA) data.’ The ‘OncoPrint’ module included ‘alteration frequency’ and ‘genetic alteration’. Based on data from the TCGA database, the UALCAN web tool (http://ualcan.path.uab.edu/index.html), a comprehensive online database, was used to analyze the expression level of SULF1 in primary and metastatic tumors. The ‘Correlation Analysis’ module in GEPIA2 analyzed the relationship between SULF1 and HER2 expressions.

### 2.5 LinkedOmics data processing

The LinkedOmics database (https://www.linkedomics.org/login.php, data obtained on 10 September 2023) is mainly used to analyze TCGA breast invasive carcinoma using a comprehensive online platform.^16^ Spearman correlation analysis of SULF1 co‐expression on the heatmap was conducted using the LinkedOmics functional module. GO, KEGG, and GSEA analyses were presented in the ‘LinkInterpreter’ module.

### 2.6 Correlation between SULF1 and drug response

The sensitivity of SULF1‐related drugs was demonstrated on RNAactDrug (http://bio‐bigdata.hrbmu.edu.cn/RNAactDrug/), an integrated database for exploring the relationship between RNA and drug sensitivity, and drugs with FDR <0.05 were selected. We also used the CPADS (https://smuonco.shinyapps.io/CADSP/) web page for analyzing drug sensitivity data from the GEO, TCGA, and GDSC databases. Based on the *p* < 0.05 significance criterion, the expression of SULF1 and concentrations of 5 chemotherapy medicines that cause 50% growth inhibition (IC50) were analyzed using the CPADS tool and the Genomics of Drug Sensitivity in Cancer (GDSC) database.

## 3 RESULTS

### 3.1 Identification of common DEGs

**FIGURE 2:**
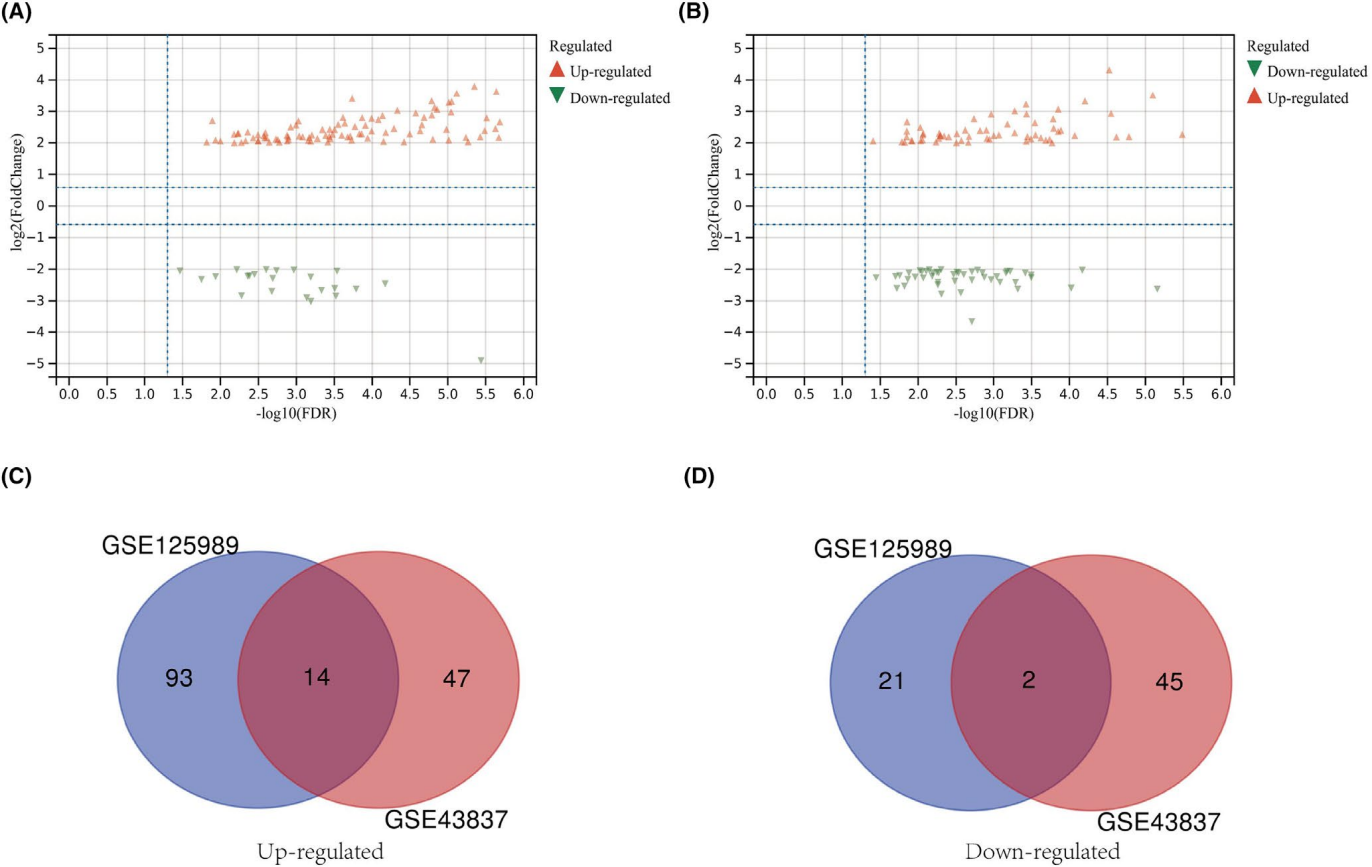
Common up‐regulated and down‐regulated differentially expressed genes (DEGs) from two datasets in the GEO database. (A) and (B), Volcano plots show co‐up‐regulated and down‐regulated genes in primary breast cancer and brain metastasis samples from GSE125985 and GSE43837. (C) and (D), Venn diagrams show co‐up‐ and down‐regulated DEGs between GSE125989 and GSE43837.

The profiles of GSE43837 and GSE125989 were analyzed separately using the online web tool GEO2R to collect DEGs common to both primary BRCA samples and BM samples. The DEGs in GSE43837 and GSE125989 were screened and shown using the Sangerbox web tool with a cut‐off criteria of |log_2_FC| ≥ 2 and a *p* value < 0.05 (Figure 2A,B), and 14 overlapping up‐regulated DEGs (47 in GSE43837 and 93 in GSE125989) and 2 overlapping down‐regulated genes (45 in GSE43837 and 21 in GSE125989) were identified (Figure 2C,D). The dataset analysis showed that the mRNA levels of GAS1, COL3A1, COL8A2, LUM, MMP13, SULF1, MME, IGK, MFAP5, COLA2, COL6A3, POSTN, COL10A1, and ASPN were significantly increased, while ATP1A2 and MBP were notably decreased.

### 3.2 Poor prognosis analyses show increased expression and alteration of SULF1 in metastatic BRCA

**FIGURE 3:**
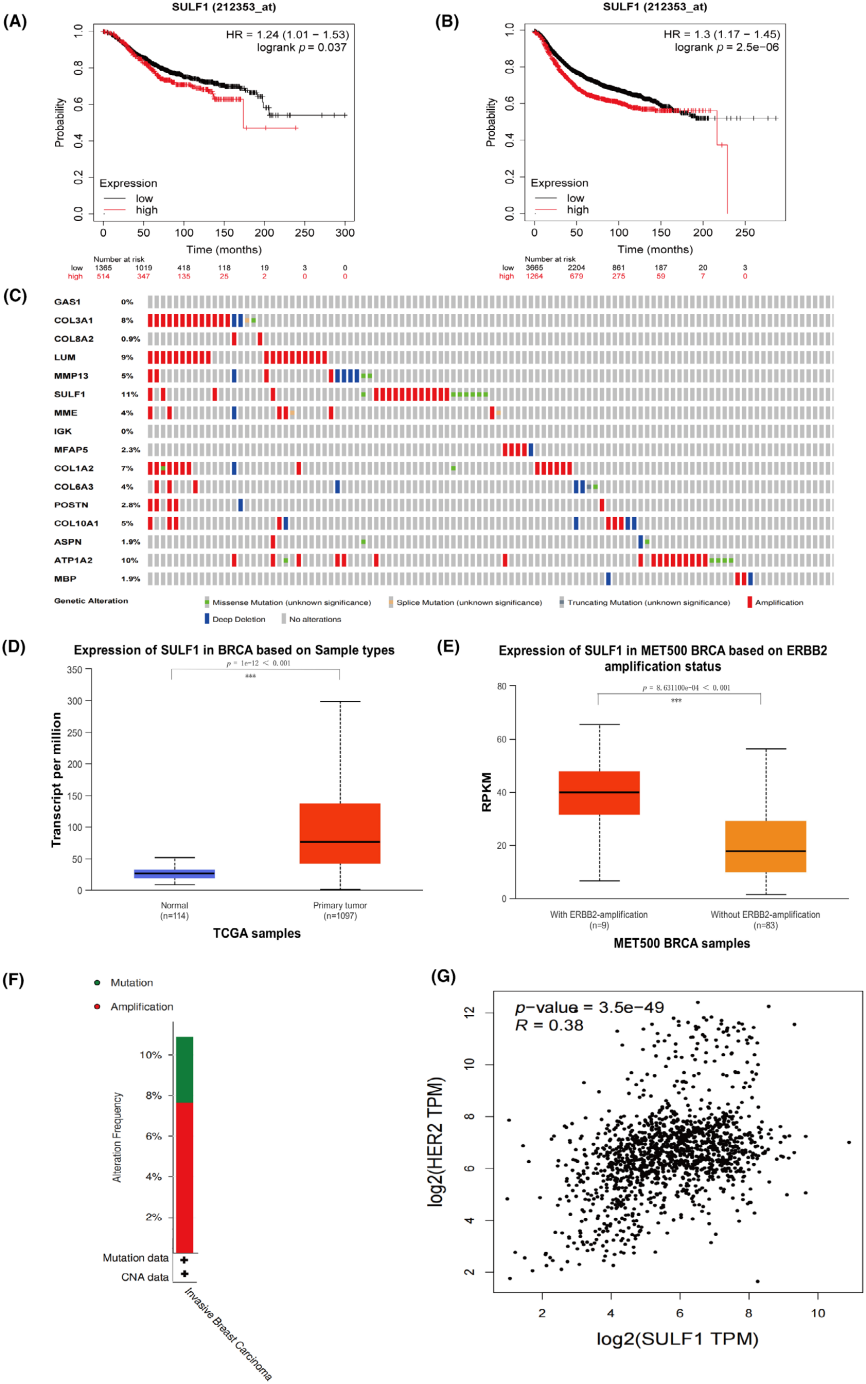
Survival prognosis, genetic variation of 16 DEGs, and the expression of SULF1. (A) and (B), OS and RFS with high expression of SULF1 of patients with breast cancer. (C), The mutation rate of 16 DEGs. (D) and (E), The mRNA expression of SULF1 in primary BRCA and metastatic BRCA samples. (F), Type and frequency of alteration of SULF1. (G), The correlation analysis between SULF1 and HER2.

Statistic analyses of OS and RFS among 16 DEGs in BRCA were conducted using the Kaplan–Meier Plotter. High‐expression and low‐expression groups were split at the best cut‐off, and the HR was calculated. Based on the *p* < 0.05 significance criterion, 16 genes were framed. The survival analyses demonstrated that only over‐expression of SULF1 was associated with unfavorable OS and RFS in BRCA patients (*p* < 0.05 for OS and PFS, Figure 3A,B). In addition, genetic and epigenetic changes closely regulated gene expression and cancer proliferation. We used cBioPortal to explore changes in 16 DEGs in metastatic breast cancer.^17^ Samples with mutation and CNA data were found in 216 patients. Consistent with the prognosis results, the mutation rate of SULF1 was the highest in the 16 DEGs (Figure 3C). These results suggested that SULF1 might be a vital factor in driving the specific brain tropism metastasis in BRCA.

Additionally, we investigated the expression of SULF1 in normal tissues and primary tumors using the UALCAN cancer database. The expression of SULF1 in primary tumors was higher than in the corresponding normal tissues (Figure 3D). Furthermore, the correlation between SULF1 expression and HER2 amplification status in breast cancer (MET500 Dataset^18^) was also screened. Expression of SULF1 was higher in metastatic breast cancer with ERBB2‐amplification (HER2‐amplification) status (Figure 3E). Considering the high expression of SULF1 in primary breast cancer and in metastatic breast cancer with ERBB2 amplification, we further investigated mutations of SULF1 in breast cancer and their relationship with ERBB2 (HER2). We investigated the mutation frequency of SULF1 in metastatic breast cancer.^17^ In 216 cases, 10.65% samples exhibited genetic alterations, of which 7.14% were amplified (Figure 3F). We also conducted gene correlation analysis on BRCA and normal tissues from TCGA datasets. The result showed that SULF1 was positively correlated with ERBB2 (HER2) (*p* < 0.05) (Figure 3G).

### 3.3 A positive correlation between ECM‐receptor and SULF1 in BRCA

**FIGURE 4:**
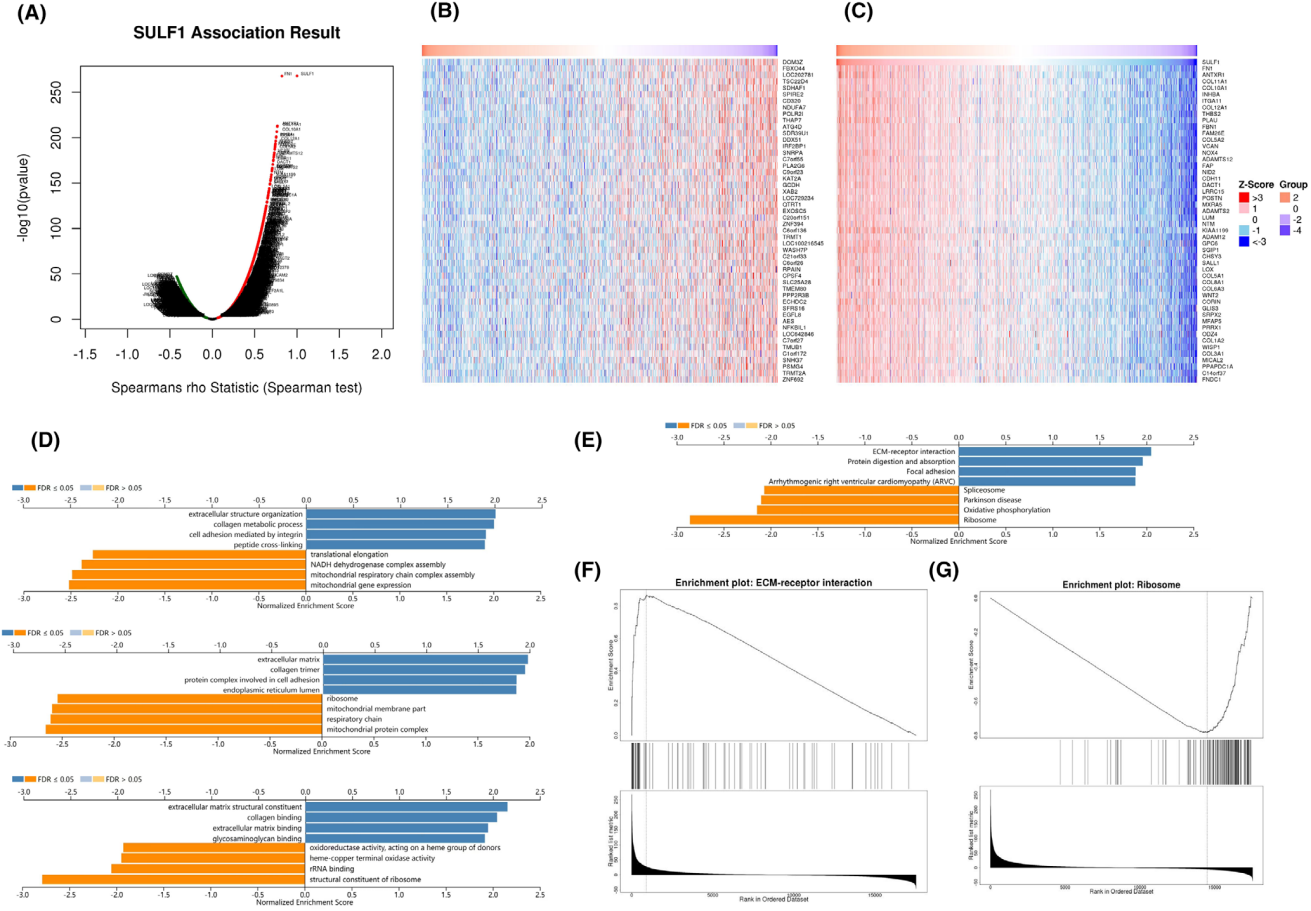
The co‐expressed genes of the SULF1 in BRCA are positively correlated with SULF1 from the LinkedOmics database. (A), The volcano plot represents the correlation between SULF1 and highly correlated DEGs identified by the Spearman test. (B) and (C), Top 50 genes with positive and negative correlations with co‐expression of SULF1. (D), Significantly enriched GO terms analysis. (E), Significantly enriched KEGG pathway analysis. (F), (G)ECM‐receptor interaction and Ribosome are differentially enriched pathways. GO, gene ontology; GSEA, gene set enrichment analysis; KEGG, Kyoto Encyclopedia of Genes and Genomes.

To better explore the mechanism of SULF1, we searched for genes co‐expressed with SULF1 within the LinkedOmics construction module. In BRCA cases, the platform chosen was HiSeq RNA in both the Search and Target datasets (ID‐146065). The volcano plot indicated that based on the significance (*p* value) of the Spearman correlation coefficient, more positively correlated genes were observed with SULF1 co‐expression than negatively correlated genes (Figure 4A). Heatmaps were used to display the top 50 genes with the strongest positive and negative correlations. DOM3Z was the top negatively correlated gene, while FN1 was the top positively correlated gene (Figure 4B,C). GO, KEGG, and GSEA were used to analyze material concentrations and genetic pathways to determine the bio‐significance and concentration pathways involved. GO analysis suggested that the genes co‐expressed with SULF1 were mainly involved in extracellular structure organization, extracellular matrix and extracellular matrix structural constituents (Figure 4D). The KEGG pathway analysis showed that the mentioned genes were associated with ECM‐receptor interaction (Figure 4E). We chose ECM‐receptor interaction and Ribosome as the terms of interest for GSEA analysis (Figure 4F,G).

### 3.4 Docetaxel and vinorelbine can act as options for treating high SULF1 expression

**FIGURE 5:**
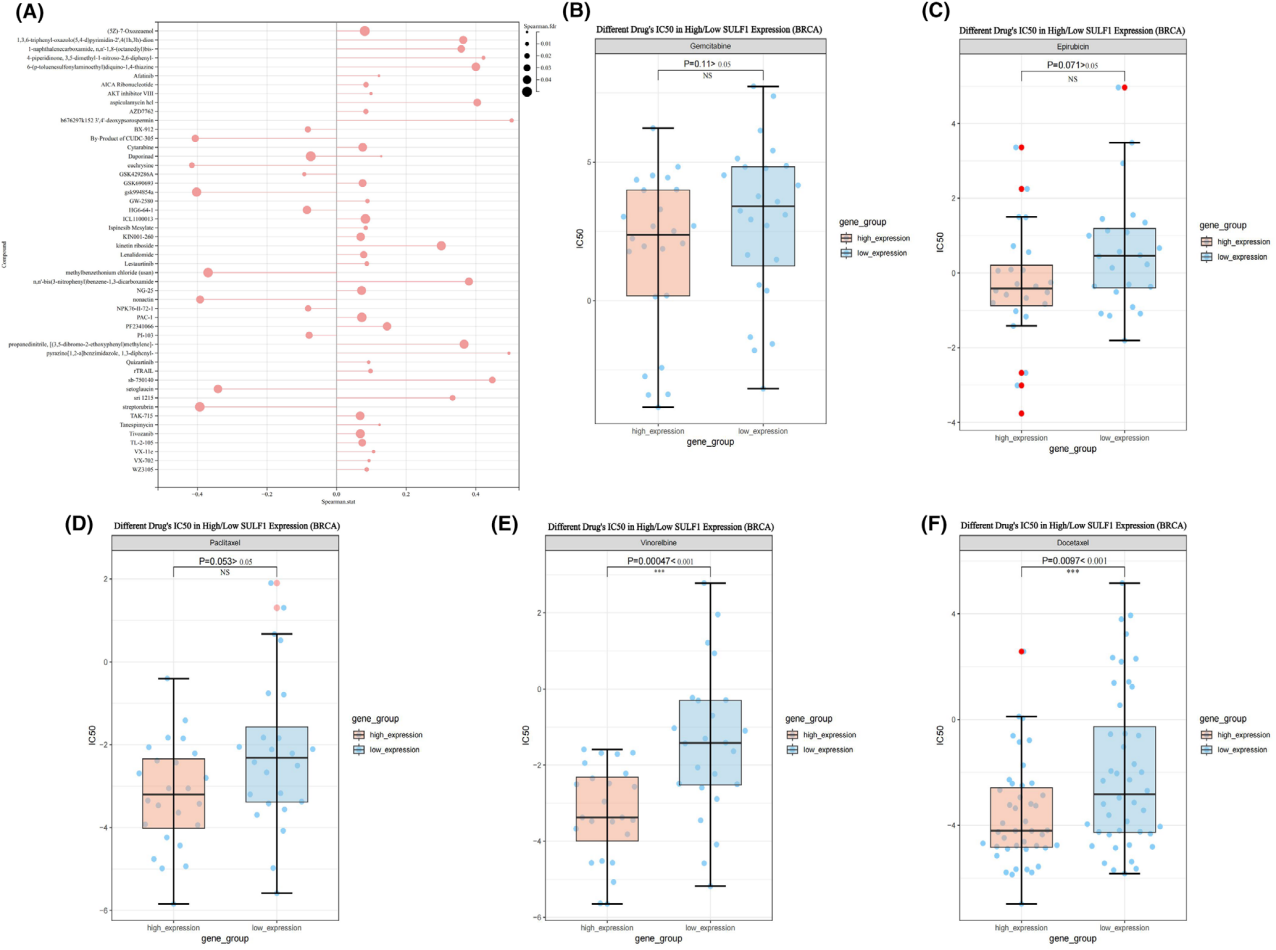
The lollipop chart and box plots show the correlation between SULF1 and drugs in RNAactDrug and CADSP. (A), The lollipop chart indicates the Spearman correlation between SULF1 and the drugs in RNAactDrug (FDR <0.05). (B–F), Box plots show antagonism of gemcitabine, epirubicin, paclitaxel, vinorelbine, and docetaxel to SULF1.

To explore potential therapeutic drugs for breast cancer brain metastasis with high SULF1 expression, we conducted searches in two drug screening databases. Firstly, we searched for drugs related to SULF1 mRNA expression in RNAactDrug. Among the top 50 drugs, the drug sensitivity of 7 drugs was significantly negatively correlated with the expression level of SULF1(FDR <0.05, stat<‐0.3), and the drug sensitivity of 12 drugs was significantly positively correlated with the expression of SULF1 (FDR <0.05, stat>0.3) (Figure 5A).

Furthermore, based on the *Breast Cancer Diagnosis and Treatment Guidelines* published by the National Health Commission of China in 2022,^19^ we screened the CPADS drug database to investigate potential target drugs for treating high SULF1 expression among the current treatment regimens for advanced breast cancer patients. A drug IC50 value that was lower in the high‐expression group than the low‐expression group indicated that the chemotherapy drug was more effective in inhibiting the high expression of SULF1. After studying drugs such as paclitaxel, docetaxel, epirubicin, epirubicin, pirarubicin, capecitabine, gemcitabine, vinorelbine, eribulin, and pertuzumab, it was found that currently, only vinorelbine and docetaxel may serve as potential drugs for high SULF1 expression (*p* < 0.05) (Figure 5B–F).

## 4 DISCUSSION

Previous research has shown that the incidence of BM is ten times higher than all primary brain tumors combined.^20^ Most BM originates from lung and breast cancer, and results in high mortality and poor prognosis.^21^ Brain metastases occur in 10%–30% of patients with metastatic breast cancer,^22^ and the median time from diagnosis of breast cancer to the discovery of brain metastases, and from the discovery of brain metastases to death, is 34 months and 15 months, respectively.^23^ Various anti‐tumor drugs are currently being studied for their efficacy in the central nervous system, but effective treatment options remain limited. Although temozolomide has high permeability through the blood–brain barrier, its effect on breast cancer brain metastasis is relatively low.^24^, ^25^ It has been proposed that the efficacy of capecitabine in the treatment of brain metastasis may be enhanced using a human concentrative nucleoside transporter (hCNT) to traverse the blood–brain barrier,^26^, ^27^ and clinical phaseIIand III trials are underway for treatment regimens involving a combination of multiple drugs with radiotherapy (NCT01077726, NCT00977379/XERAD, NCT00570908). Other fundamental and clinical trials are also underway. Bioinformatics analysis uses existing transcriptome data and clinical data to quickly predict and analyze potential targets, thus avoiding long periods of experimental time, and has become a critical tool in scientific research. For example, studies have identified breast cancer mutation genes using multiple omics data using the MutsigCV algorithm,^28^ and established a breast cancer subtype prediction model of NPI through a deep learning model.^29^ Considering the high incidence of BM and recent progress in research and treatment options, exploring the related molecular mechanisms of breast cancer brain metastasis, discovering potential therapeutic targets, and understanding the prognosis of advanced breast cancer are of paramount importance.

In this study, we identified 16 DEGs by searching two different datasets of breast cancer brain metastasis (GSE43837 and GSE125989). Among these 16 DEGs, high expression of SULF1 had the highest gene mutation rate, reaching up to 11%, and was found to shorten the OS and RFS of breast cancer patients. Furthermore, in the subgroup of metastatic breast cancer patients with HER2 amplification, high expression of SULF1 was observed. Correlation analysis between HER2 and SULF1 showed a positive relationship. Previous studies have confirmed the high incidence of breast cancer brain metastasis, especially in HER2‐positive and triple‐negative subtypes, with rates as high as 30%–40% and a one‐year survival rate of only 20% after metastasis.^30^, ^31^, ^32^ The relationship between SULF1 and HER2 is still being determined, and is the subject of further investigation.

It has been reported that there is a significant correlation between SULF1 and BRCA risk, with high SULF1 expression in BRCA tissues, which may promote tumor growth and metastasis.^32^, ^33^, ^34^ Short SULF1 splice variants were predominant in breast tumors, promoting the growth of MDA‐MB231 and MCF7 cell lines derived from breast tumors induced by Fgf2 in vitro. Some studies have suggested that inhibiting SULF1 may target tumor cells by down‐regulating the RTK pathway.^32^

We obtained a list of genes related to breast cancer metastasis associated with SULF1 from the GO and KEGG databases, revealing an association between the SULF1 gene and the ‘extracellular matrix’ (ECM). Increased secretion of SULF1 may enhance the remodeling of the extracellular matrix in the tumor microenvironment, affecting the development of tumors and adjacent host cells.^35^ During normal morphogenesis, mammary ducts surround a stroma composed of ECM and various cell types.^36^ The ECM, a major mammary microenvironment component, regulates the structure and function of epithelial cells. Focal adhesion, invasion, and metastasis of tumors depend on the remodeling, stiffening, and degradation of ECM in the tumor microenvironment.^37^, ^38^ In addition, many studies have confirmed the relationship between malignant transformation and metastasis and cell adhesion processes. Cell‐matrix adhesion includes cell movement, proliferation, differentiation, regulation of gene expression, and survival, all of which are crucial biological processes.^39^ Therefore, in breast cancer cells, SULF1 may play a role in malignant transformation by participating in the cell‐matrix adhesion process, leading to cell adhesion imbalance, changes in the tumor microenvironment, and ultimately, disruption of the blood–brain barrier, resulting in breast cancer brain metastasis.^8^

According to the current chemotherapy regimens for advanced breast cancer patients, we matched the expression of SULF1 with the sensitivity of first‐line drugs. The results showed that when SULF1 was highly expressed, the IC50 values for docetaxel and vinorelbine were significantly reduced. These results suggest that these two drugs may be potential targets as chemotherapy drugs acting against SULF1.

In conclusion, using bioinformatic analysis, we propose that SULF1 can serve as a prognostic marker for poor outcomes in breast cancer brain metastasis and is a potential therapeutic target. Additionally, we analyzed the possible mechanisms of breast cancer brain metastasis and the pathways in which SULF1 was involved. Further experimental and clinical trials investigating the role of SULF1 in breast cancer brain metastasis should be beneficial in understanding breast cancer treatment and providing new possibilities for more treatments.

## AUTHOR CONTRIBUTIONS

All listed authors meet the requirements for authorship. Y.T. Li conceived the research, analyzed the data, and wrote the article's first draft. W.L. Zhang revised the article. Q. Kong supervised the project. J. Wang and Y. Wu searched for information. T.T. Feng and Q.H. Wang searched for data. All authors read and approved the final manuscript.

## FUNDING INFORMATION

The Special Research Fund for Central Universities, Peking Union Medical College (No. 3332022182).

## CONFLICT OF INTEREST STATEMENT

The authors report no conflicts of interest.

## ETHICS STATEMENT

All the data were retrieved and available online, and this research did not involve any experiments on humans or animals. Furthermore, all informed consents had been obtained.
